# Long term follow-up and liver-related death rate in patients with non-alcoholic and alcoholic related fatty liver disease

**DOI:** 10.1186/1471-230X-14-166

**Published:** 2014-09-27

**Authors:** Svanhildur Haflidadottir, Jon G Jonasson, Helga Norland, Sylvia O Einarsdottir, David E Kleiner, Sigrun H Lund, Einar S Björnsson

**Affiliations:** Department of Internal Medicine, Section of Gastroenterology and Hepatology, The National University Hospital of Iceland, Hringbraut 11D, 101 Reykjavik, Iceland; Department of Pathology, The National University Hospital, Reykjavík, Iceland; Faculty of Medicine, University of Iceland, Reykjavík, Iceland; Laboratory of Pathology, NIH/NCI, Bethesda, MD USA; Centre of Public Health, University of Iceland, Reykjavík, Iceland

**Keywords:** NAFLD, AFLD, Fibrosis, Prognosis, Mortality

## Abstract

**Background:**

Few studies have compared the prognosis and liver-related mortality in patients with NAFLD (nonalcoholic fatty liver disease) and AFLD (alcoholic fatty liver disease). We aimed to investigate the etiology and liver-related mortality of patients with liver biopsy verified fatty liver disease in a population based setting.

**Methods:**

In this retrospective study, all patients who underwent a liver biopsy 1984–2009 at the National University Hospital of Iceland were identified through a computerized pathological database with the code for fatty liver. Only patients with NAFLD and AFLD were included and medical records reviewed. The patients were linked to the Hospital Discharge Register, the Causes of Death Registry and Centre for Addiction Medicine.

**Results:**

A total of 151 had NAFLD and 94 AFLD with median survival of 24 years and 20 years, respectively (p = NS). A total of 10/151 (7%) patients developed cirrhosis in the NAFLD group and 19/94 (20%) in AFLD group (p = 0.03). The most common cause of death in the NAFLD group was cardiovascular disease (48%). Liver disease was the most common cause of death in the AFLD group (36%), whereas liver-related death occurred in 7% of the NAFLD group. The mean liver-related death rate among the general population during the study period was 0.1% of all deaths. There was a significantly worse survival for patients in the AFLD group compared to the NAFLD group after adjusting for gender, calendar year of diagnosis and age at diagnosis (HR 2.16, p = 0.009). The survival for patients with moderate to severe fibrosis was significantly worse than for patients with mild fibrosis after adjusting for gender, calendar year of diagnosis and age at diagnosis (HR 2.09, p = 0.01).

**Conclusions:**

Patients with fatty liver disease showed a markedly higher risk of developing liver-related death compared to the general population. The AFLD group had higher liver-related mortality and had a worse survival than the NAFLD group. Patients with more severe fibrosis at baseline showed a worse survival than patients with none or mild fibrosis at baseline.

## Background

Fatty liver disease is clinically categorized into two main groups, alcoholic fatty liver disease (AFLD) and non-alcoholic fatty liver disease (NAFLD). NAFLD is becoming one of the most common liver diseases worldwide [1] with a prevalence up to 30% in the general population [2] and it can progress to end-stage liver disease [3]. NAFLD is associated with insulin resistance, and has been considered to be the hepatic component of the metabolic syndrome [3–5]. Patients with NAFLD have been shown to have increased cardiovascular mortality compared to the general population [6, 7]. Only a few studies have assessed the prognosis and risk of liver-related death in patients with biopsy verified NAFLD in a population based setting [6, 7]. Thus, even though NAFLD is potentially a serious condition well designed population based studies on its natural history are lacking. Most recent studies on fatty liver disease have focused on NAFLD although AFLD is an important cause of fatty liver and only a few studies have compared the long term prognosis between NAFLD and AFLD [8–10].

The aims of this study were 1) to examine the natural history and outcome of patients with fatty liver disease with non-alcoholic and alcoholic etiologies*.* 2) Compare the prognosis and liver-related mortality in patients with NAFLD and AFLD, and compare these patients to the liver-related mortality in the general population. Our hypothesis was that these patients suffer from increased liver-related morbidity compared to the general population.

## Methods

### Population and case finding

In this retrospective study, a search was undertaken in a computerized diagnoses database from 1984 (when the pathology registry commenced their electronic registration) to 2009, in the Department of Pathology at the National University Hospital (NUH) of Iceland and identified all liver biopsies analysed and registrated in the SNOMED coding-system, T-56000 and the M-50080 as having fatty change. The SNOMED (Systemized Nomenclature of Medicine) is a coding system used in the pathology laboratories in Iceland to specify: Procedure, Topography, Morphology, Disease and Etiology. This is a very valuable coding system for retrieving data and pathology reports from past years, like biopsies of the liver (T-56…) showing fatty change (M-50080).

The catchment area of the NUH covers >95% of the population in Iceland.

All medical records from these patients were examined with respect to the following exclusion criteria: 1) presence of acute or chronic liver disease: PBC, autoimmune hepatitis, alfa-1-antitrypsin deficiency, hemochromatosis and viral hepatitis. 2) jejunoileal bypass operation. 3) use of drugs known to be associated with fatty liver disease such as methotrexate, amiodarone, tamoxifen and high doses of corticosteroids. 4) malignancy at the time of index liver biopsy. 5) age under eighteen years at the time of index liver biopsy. 6) gallstone surgery at the time of index liver biopsy. The patients with gallstone surgery at the time of index liver biopsy were excluded to better represent the patients who would undergo a liver biopsy in clinical practice and not just the incidental finding of fatty liver during an operation.

**Figure 1:**
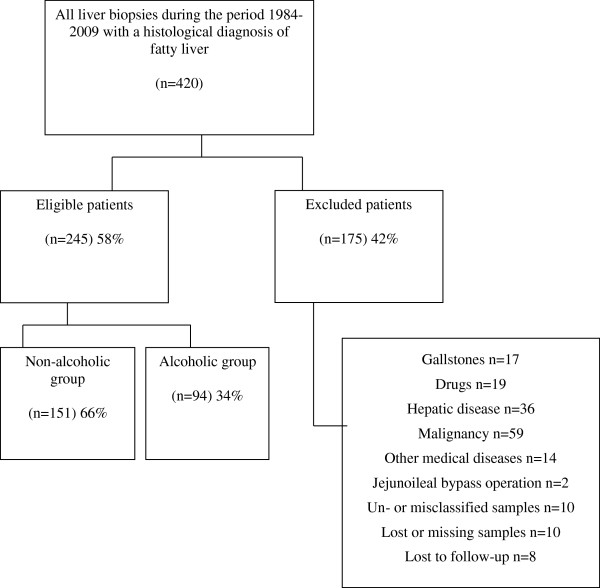
**Flowchart of the patients.** Reasons for exclusion from the study are shown.

Indications for the index biopsy in the cohort were elevated liver tests, mainly serum alanine aminotransferase (ALT) and serum aspartate aminotransferase (AST) and/or hepatomegaly or suspected alcoholic liver disease. A total of 420 patients met the inclusion criteria and were divided into two groups, non-alcoholic and alcoholic group respectively (Figure 1).

### Data collection

The following information was obtained from the medical records and recorded at baseline: gender, age at diagnosis, height, weight and body mass index (BMI), history of diabetes mellitus, hyperlipidemia, hypertension, cardio- and cerebrovascular disease, liver disease and malignancy. Data on drug and alcohol intake was noted in the medical records. Patient with a reported alcohol problem or abuse or an alcohol-related diagnosis before or at the time of liver index biopsy were considered to have alcoholic fatty liver disease. The diagnosis of cirrhosis was accepted in patients who had a discharge diagnosis, a death certificate diagnosis and/or histological confirmation in the follow-up period indicating cirrhosis. Laboratory data included at baseline were: AST, ALT, bilirubin, albumin, alkaline phosphatase (ALP), prothrombin time (PT), glucose, platelets count (PLT), serum cholesterol, serum triglycerides (TG) and mean corpuscular volume (MCV). The last follow-up date was the 30th of November 2011.

### Follow-up

All members of the cohort were linked through their unique personal identification number to the centralized Hospital Discharge Register at the Directorate of Health and the Registry of Causes of Death (RCD) at Statistics Iceland. Information was obtained from National Centre of Addiction Medicine in Iceland on whether or not the patients had undergone addiction therapy before or after the time of the index liver biopsy. This information was obtained from the addiction centre without knowledge of the liver biopsy results.

Patients were excluded if they were lost to follow-up in the registries. 8 patients were lost to follow-up.

### Ethical considerations

The study was approved by the National Bioethics Committee (10-029-V1-S1) and the Icelandic Data Protection Authority (S4766/2010).

### Histological classification

The index liver biopsies were formalin fixed and treated routinely in the pathology laboratory. They were paraffin embedded and cut in 4–5 micrometer thick sections. The sections were stained with hematoxylin and eosin, periodic acid Schiff reagent (PAS) with diastase and for reticulin. In addition a connective tissue stain, Weigert van Gieson or most commonly Masson Trichrome stain was performed. All histological slides were reviewed by experienced pathologists without knowledge of the clinical or biochemical data of the patients. Three pathologists (Jon G. Jonasson, David E. Kleiner and Sylvia O. Einarsdottir) divided the slides between them and reviewed all of the samples over a period of 2 years. The assessment was blinded to the clinical details. Second review to examine inter-observer variability was not undertaken. Morphological findings were recorded in a semi-quantitative manner regarding steatosis and fibrosis, which was initially proposed by Brunt et al. in 1999 and developed further by Kleiner et al. [11, 12]. Grade 0 steatosis was defined as < 5% fat and thus not compatible with fatty liver disease. Fibrosis stage were defined as follow: 0: none. 1: Perisinusoidal or periportal fibrosis. 1A: mild, zone 3, perisinusoidal. 1B: Moderate, zone 3, perisinusoidal. 1C: Portal/periportal. 2: Perisinusoidal, portal/periportal. 3: Bridging fibrosis. 4: Cirrhosis.

### Statistical analysis

Statistical testing was performed using R-software. The results were presented as medians, interquartile range and range and as number (%). The Mann–Whitney *U*-test was used to test for differences between groups. Dichotomous variables were compared by using the Chi-square (*X*^2^) or Fischer’s exact test. A p-value of less than 0.05 was considered statistically significant. The survival curves were estimated by the Kaplan-Meier estimator, using years as the time-scale and taking delayed entry into account. Differences between groups were investigated by Wald test in Cox proportional hazards model, with age as the time-scale, adjusting for gender, calendar year of diagnosis and age of diagnosis. The primary end-point was death from all causes.

## Results

Overall 2262 liver biopsies were performed at the University Hospital of Iceland during the study period. A total of 420 patients with at least one index biopsy were identified by the computerized search in the pathology database as having fatty liver on biopsy. Those who were misclassified and did not have fatty liver on review were excluded (Figure 1). A total of 175 patients were excluded for other reasons than based on histopathology (Figure 1). The remaining study group of 245 patients had no signs of viral hepatitis in the index liver biopsy and did not receive any medication known to be associated with the development of steatosis.

Medical records from these patients were traced and the biopsies reviewed by experienced pathologists. A total of 245 patients constituted the study group, 136 (56%) women and 109 men (44%). Women were in the majority in the NAFLD group, 105/151 (70%) compared to 46/151 (30%) men (p < 0.001). The proportion of men was higher in the AFLD group, that is 67% (63/94) whereas the proportion of women was 31/94 (33%).

### Clinical and biochemical results

Clinical and biochemical data at the time of index liver biopsy in the 245 patients of the two study groups (Table 1). Information was available to calculate BMI in 56% of the total study cohort. No significant difference was found in the BMI between the two groups (Table 1). In the current context obesity was defined as BMI ≥ 30, 48/88 patients (55%) were obese in the NAFLD group compared to 22/50 patients (44%) in the AFLD group (NS). In the total cohort no significant difference was observed between the genders in terms of obesity as 31/76 (41%) of women were obese and 31/62 (50%) of the men and no difference was seen in BMI when comparing genders in the NAFLD and the AFLD group (data not shown). A total of 74/88 (84%), 52 women vs. 22 men in the NAFLD group had a BMI > 25 compared to 38/50 (76%), 9 women vs. 29 men in the AFLD group (NS).

**Table 1:** **Clinical and biochemical data at the time of index liver biopsy**

Baseline			End of follow-up			
	NAFLD group	AFLD group	p-value	NAFLD group	AFLD group	p-value
	n = 151	n = 94		n = 151	n = 94	
Gender (F/M)	105/46	31/63	<0.001			
Female%	70%	33%				
	Mean (sd) or Median (IQR)	Mean (sd) or Median (IQR)				
Age (years)	54 (14.7)	51 (13.5)	NS			
BMI (kg/m^2^)	29 (26–32)	29 (25–32)	NS			
AST (U/L)	47 (32–63)	76 (44–157)	<0.001	48 (36; 28–46)	91 (46; 28–93)	0.05
ALT (U/L)	69 (44–102)	109 (58–198)	0.001	44 (36; 26–52)	67 (45; 27–75)	0.04
Bilirubin (μmol/L)	10 (7-15)	12 (9-21)	0.01	14 (9; 7–18)	49 (11; 9–21)	NS
ALP (U/L)	197 (135–320)	179 (134–280)	NS	196 (122; 87–157)	172 (119; 77–179)	NS
Prothrombin time (sec)	13 (12-14)	14 (13-15)	0.02	16 (14; 13–17)	16 (14; 13–17)	NS
Albumin (g/L)	40 (36–44)	39 (32–41)	NS	37 (39; 33–42)	35 (37; 28–41)	NS
Platelets (x10^9^/L)	265 (92.9)	228 (83.3)	0.006	235 (238; 170–281)	219 (222; 152–265)	NS
Random blood glucose (mmol/L)	6 (5-7)	6 (5-7)	NS	7 (6; 5–7)	7 (6; 5–7)	NS
Cholesterol (mmol/L)	6 (5,6)	5 (5-7)	NS	5 (5; 4–6)	5(5; 4–6)	NS
Triglycerides (mmol/L)	2 (1-3)	2 (1-3)	NS	2 (2; 1–2)	2 (2; 1–2)	NS
MCV (fL)	90 (6.1)	95 (7.2)	<0.001	90 (90; 87–94)	95 (95; 90–105)	<0.001

*Abbreviations:* *F* female, *M* male, *BMI* body mass index, *AST* aspartate aminotransferase, *ALT* alanine transaminase, *ALP* alkaline phosphatase, *MCV* mean corpuscular volume, *NS* not significant. Mean (sd) is shown for normally distributed variables, but median (IQR) for other variables. Obesity was defined as BMI ≥ 30 (kg/m).

The biochemical markers AST, ALT, bilirubin, prothrombin time and MCV were higher and platelets lower in the AFLD group compared to the NAFLD group at the time of index liver biopsy whereas other biochemical markers were similar in the two groups (Table 1**).** At the end of follow-up, ALT, AST and MCV were the only biochemical markers still significantly higher in the AFLD group compared to the NAFLD group (Table 1).

The clinical data on other diseases associated with the metabolic syndrome at the time of index liver biopsy and at the end of follow-up period in the two study groups are shown in Table 2. The two groups had also similar morbidity in terms of conditions associated with metabolic syndrome both at baseline and at follow-up (Table 2). Overall, 58 (62%) of the patients in the AFLD group had undergone alcohol addiction therapy according to computerized database of patients in the National Centre of Addiction Medicine, whereas 2 (1%) in the NAFLD group had undergone an addiction therapy, not due to alcohol but due to abuse of sedatives and due to a gambling addiction.

**Table 2:** **Comorbid diseases at baseline and the end of follow-up period**

Baseline				End of follow-up		
	NAFLD	AFLD	p-value	NAFLD	AFLD	p-value
	n = 151	n = 94		n = 151	n = 94	
	n (%)	n (%)		n (%)	n (%)	
DM II	22 (15)	8 (9)	NS	42 (28)	16 (17)	NS
HTN	53 (35)	37 (39)	NS	79 (52)	44 (47)	NS
Hyperlipidemia	28 (19)	12 (13)	NS	32 (21)	16 (17)	NS
Cardio- and cerebrovascular disease	19 (13)	11 (11)	NS	44 (29)	24 (26)	NS

*Abbreviations:* *NAFLD* non-alcoholic fatty liver disease, *AFLD* alcoholic fatty liver disease, *DM II* diabetes mellitus type II, *HTN* hypertension.

### Histological end-points and development of cirrhosis

The histological characteristics in the index liver biopsy are summarized in Table 3. According to the NAS score overall 31/151 (21%) patients in the NAFLD group had NASH compared to 35/94 (37%) with ASH in the AFLD group (p = 0.007). In the nonalcoholic group 47 (31%) patients had borderline NASH and 68 patients (45%) did not have steatohepatitis. In the alcoholic group 35 (37%) patients had borderline ASH (NS) and 22 (23%) did not have ASH (NS). Patients in the NAFLD had less severe lobular inflammation than the AFLD group (Table 3).

**Table 3:** **Histological characteristics of NAFLD- and AFLD group at index biopsy**

	NAFLD group	AFLD group	p-value
	n (%)	n (%)	
**Steatosis**			
1 (5-33%)	77 (51)	37 (39)	NS
2 + 3 (≥33%)	74 (49)	57 (61)	NS
**Lobular inflammation**			
0 + 1 (no foci or <2 foci/200x)	99 (66)	43 (46)	0.003
2 + 3 (2–4 foci or >4 foci/200x)	52 (34)	51 (54)	0.003
**Ballooning**			
0 + 1 (none or few balloon cells)	139 (92)	90 (96)	NS
2 (many cells)	12 (8)	4 (4)	NS
**Fibrosis**			
0 + 1 + 1A + 1B + 1C (none to mild fibrosis)	120 (79)	61 (65)	0.02
2 + 3 + 4 (moderate to severe)	31 (21)	33 (35)	0.02
**NAS score**			
<3	68 (45)	22 (23)	0.001
3-4	47 (31)	35 (37)	NS
≥5	31 (21)	35 (37)	0.007

At the time of index liver biopsy 18 patients had cirrhosis, 6 patients in the NAFLD group and 12 in the AFLD group, respectively. Overall, 11 patients developed cirrhosis during follow-up period, four patients in the NAFLD group and seven patients in the AFLD group. Thus, a total of 29 patients were diagnosed with cirrhosis in the two groups, 10 (7%) patients in the NAFLD group and 19 (20%) patients in the AFLD group (p = 0.003).

Among patients developing cirrhosis in the follow-up period the histological diagnosis at baseline was as follow: one patient had no fibrosis (in the NAFLD group), three patients had stage 1A fibrosis (all three in the AFLD group), one patient had portal fibrosis (in the NAFLD group) and six patients bridging fibrosis (four in the AFLD group and two in the NAFLD group).

### Liver-related complications

Patients developing liver cirrhosis and liver related complications are demonstrated in Table 4. Among patients diagnosed with cirrhosis a somewhat higher proportion developed ascites in the AFLD group, 11/19 (58%) vs. NAFLD, 3/10 (30%) (p = 0.004). Only one patient in the AFLD group developed HCC but none of the NAFLD patients developed HCC (Table 4). A significantly higher number of patients in the AFLD group, 13/94 (14%) developed decompensated liver disease compared with 7/151 (5%) in the NAFLD group (Table 4). It should be noted that baseline NAS score was different between two groups with higher baseline NAS score in the AFLD group (NAS score > 5: 37% vs 21%).

**Table 4:** **Development of chronic liver disease such as HCC, portal hypertension, varices and ascites**

	NAFLD group	AFLD group	p-value
	n = 151	n = 94	
	(%)	(%)	
Cirrhosis	10 (7)	19 (20)	0.003
Death	3 (2)	9 (10)	0.02
Ascites	3 (2)	11 (12)	0.004
Varices	3 (2)	6 (6)	NS
Bleeding varices	1 (1)	1 (1)	NS
Portal hypertension	3 (2)	2 (2)	NS
HCC	0 (0)	1 (1)	NS

### Survival and mortality

**Figure 2:**
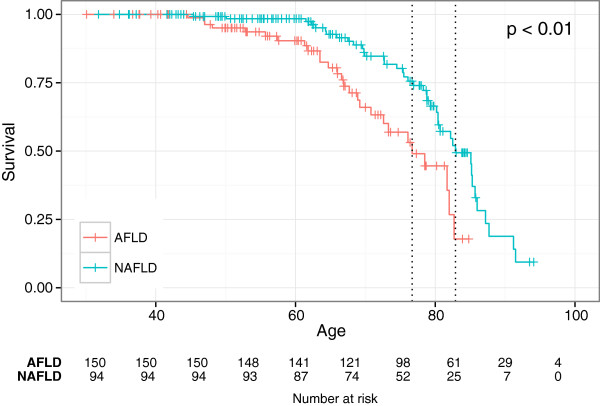
**Kaplan Meier survival plots showing survival from day of birth after correcting for age, gender and year of diagnosis for the NAFLD- and AFLD groups.** The green line showing the NAFLD (non-alcoholic fatty liver disease) group and the red line showing the AFLD (alcoholic fatty liver disease) group.

The median survival was 24.2 (range 0.2-26.1) years in the NAFLD group and 19.5 (range 0.2-24.2) years in the AFLD group (p = 0.0007). Median follow-up time for the non-alcoholic group was 9.9 years (range 0.2-26 years) and 9.2 years (0.2-25 years) for the alcoholic group. There was no significant difference in overall survival between the two study groups and no significant difference between genders (data not shown). Patients in the AFLD group diagnosed with cirrhosis had a higher death rate compared to the NAFLD group; 10 patients (40%) in the AFLD group compared with 7 patients (17%) in the NAFLD group (NS). Using Cox analysis the survival was significantly worse for patients in the AFLD group compared to the NAFLD group after adjusting for gender, calendar year of diagnosis and age at diagnosis (HR 2.16, p = 0.009) (Figure 2). The hazard ratio for women in the AFLD group was 1.19 compared to the NAFDL group.

**Figure 3:**
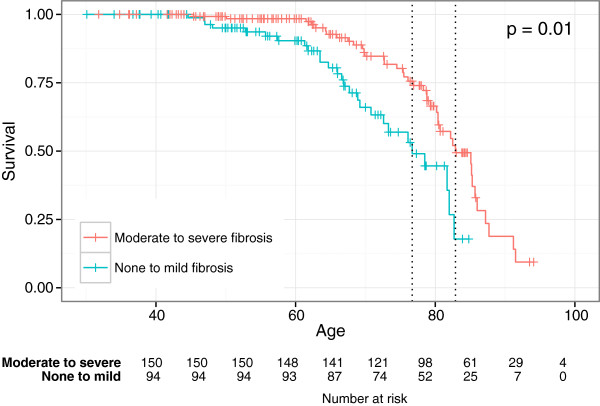
**Kaplan Meier survival plots showing survival from day of birth after correcting for age, gender and year of diagnosis for the none to mild fibrosis group (green line) and moderate to severe fibrosis group (red line).**

The survival for patients with moderate to severe fibrosis was significantly worse than for patients with mild fibrosis after adjusting for gender, calendar year of diagnosis and age at diagnosis (HR 2.09, p = 0.01) (Figure 3).

A total of 67 patients died during the follow-up period; 41 women (61%) and 26 men (39%). Of these 12 (18%) were liver related in both the NAFLD and AFLD group (Table 5). The most common cause of death was due to cardiovascular disease 28/67 (42%) followed by liver-related disease 12/67 (18%) and malignancy 12/67 (18%) (Table 5). 7% of deaths were liver-related in the NAFLD group, no man in the NAFLD group died of liver-related disease whereas three women had a liver-related death. In the AFLD group the most common cause of death was liver-related, 9/25 (36%) followed by cardiovascular disease in 8/25 (32%) and malignancy among 6/25 (24%). The mean liver-related death rate among the general population during the study period was 0.1% of all deaths [13]. In the AFLD group six men and three women died of liver-related disease. Only one patient died from hepatocellular cancer (HCC) in the total cohort and as mentioned above, from the AFLD group.

**Table 5:** **Causes of death in NAFLD- and AFLD group**

	NAFLD group (n = 42)		AFLD group (n = 25)		Total
	n (%)		n (%)		n (%)
	Men	Women	Men	Women	
Cardio- and cerebrovascular diseases	3 (15)	17 (85)	7 (88)	1 (12)	28 (42)
Liver-related diseases	0 (0)	3 (100)	6 (67)	3 (33)	12 (18)
Malignancy	2 (33)	4 (67)	3 (50)	3 (50)	12 (18)
Injury/poisoning	2 (67)	1(33)	2 (100)	0 (0)	5 (7.5)
Other chronic medical condition	1 (25)	3 (75)	0 (0)	1 (100)	5 (7.5)
ARDS/pneumonia	0 (0)	2 (100)	0 (0)	0 (0)	2 (3)
Other	0 (0)	2 (100)	0 (0)	1 (100)	3 (4.5)

The most common cause of death in the NAFLD group was of cardiovascular disease 20/42 (48%), followed by malignancy 6/42 (14%) and other chronic medical conditions 4/42 (9.5%). A total of 12 patients (18%) died of malignancy. In the AFLD group, 4 died of breast cancer and one each of renal cancer, malignant brain tumor, colon cancer, nasopharyngeal cancer and pancreas cancer. In the NAFLD group, two died of breast cancer and one each of prostate cancer, multiple myeloma, endometrial cancer and one of small cell lung cancer.

## Discussion

Few studies have determined the natural history of biopsy-proven fatty liver disease and compared the long-term prognosis of these two major groups of fatty liver disease due to alcoholic and non-alcoholic fatty liver disease.

Our study has several methodological strengths. First, all patients had biopsy proven fatty liver disease and histology was re-evaluated based on validated scores. We believe we have included the vast majority of patients with biopsy-proven fatty liver disease in the whole population of Iceland during this period as at least 95% of the population lives in the catchment area of the National University Hospital**.** The fact that all the patients underwent a liver biopsy is also a weakness, especially when evaluating disease outcome. Previous studies have shown that NAFLD patients diagnosed with liver biopsy have a worse prognosis compared with patients diagnosed with ultrasonography. Therefore, studying patients recruited from the pathology registry involves a selection bias. It must be stressed that the indications for the liver biopsy was not always clear, although most cases were because of elevated liver function tests and/or hepatomegaly or suspected alcoholic liver disease, and this in turn can give a selection bias. Also the indication for biopsy in NAFLD and AFLD may differ between conditions and between practitioners and might explain some of the differences in disease outcome.

The main limitation of the study was its retrospective design and data was not systematically registrered and was therefore sometimes missing or unavailable. In addition the search for the code M-50080 (fatty liver) is limiting in itself as the more serious steatosis with fibrosis and even cirrhosis might be coded as something else than just fatty liver. Since this was a retrospective study on liver biopsies it is not possible to standardize the size of the needle biopsies. Therefore the size is very variable and the range can be considerable, but should be similar to the standards observed in pathology departments in general. Samples less than 2 mm in diameter would have been excluded from the study, but no such samples came into the study.

The slides used for pathological estimation were the original slides and recuts or restaining of slides was not done except for a few exceptional cases where the original slides were unavailable or when a Masson-Trichrome stain had not been performed originally. Occationally the colours of the slides had faided somewhat. This however we do not anticipate having significant effect on the results, especially since ballooning degeneration increases cell size and this is not greatly affected by fading colours.

Another limitation is the small number of hard endpoints with only four patients in the NAFLD group who developed cirrhosis over the follow-up period. There is also a potential uncertainty in the non-histological diagnosis of cirrhosis which must be taken into consideration when reviewing the results.

The information on ASH should be interpreted with caution as NAFLD activity score has to our knowledge not yet been validated in AFLD, but we chose to use it for comparison as the histolopathological development is similar in the two conditions and there is no difference morphologically between NAFLD and AFLD.

One of the main findings in this study was that patients with fatty liver disease showed a markedly higher risk of developing liver-related death compared to the general population. Although significantly higher in the AFLD group liver-related death in the NAFLD group was 7% of all deaths. These findings are in contrast with liver-related death rate in Iceland [13] which was a mean of 0.1% in the general population during the study period. As in other studies it is a challenge to classify patients into non-alcoholic and alcoholic group. We tried to minimize the misclassification by regrouping those without a known alcohol etiology if the patients were found to have an alcoholic related diagnosis later as for instance alcohol pancreatitis and alcohol dependence. In addition all patients were linked to the database for the National Centre of Addiction Medicine in Iceland.

### Liver-related morbidity and mortality

Our results show that patients with NAFLD had a rather benign course in terms of liver-related morbidity and mortality. Only 7% developed cirrhosis after a mean of 13 years of follow-up which is similiar to what previous studies on the prognosis of NAFLD have shown [6, 7, 14–17]. A higher number of patients in the AFLD group developing cirrhosis (20%) after approximately 12 years of follow-up, is also in agreement with previous studies showing worse prognosis in patients with AFLD than NAFLD [8, 9, 18]. These studies have demonstrated that patients with alcoholic fatty liver disease have worse prognosis of their liver disease than patients with NAFLD [8, 9, 18]. In a study on prognosis and life expectancy in chronic liver disease the five year survival was 38% for the alcoholic group and 68% for the non-alcoholic group but only 87% of the patient underwent liver biopsy whereas the rest was diagnosed clinically or with ultrasound [18]. In another study of 7000 patients discharged with the diagnosis fatty liver the mortality was 5.4 fold amongst AFLD and 2.6 amongst NAFLD [19]. In the current study we found that the overall survival was worse in the AFLD group. Patients in the AFLD group had a higher liver-related mortality, but patients in the NAFLD group died more frequently from cardiovascular disease as already demonstrated in previous studies [6, 7, 14, 16].

In the AFLD group the most common cause of death was liver-related (35%).

Other studies have shown that obesity in both NAFLD and AFLD predispose to the development of fatty liver and chronic liver disease [19, 20]. In Iceland, the prevalence of liver cirrhosis due to alcohol is very low, only 3.3/100.000 inhabitants which was 4 times lower than in Sweden [21]. In the current study 45% of the patients in the NAFLD group had a BMI ≥30 and somewhat surprisingly there was no significant difference in BMI between the NAFLD group and the AFLD group. The fact that patients with AFLD did not differ with respect to BMI and incidence of metabolic syndrome-related diseases might reflect a mixed AFLD/NAFLD etiology in the alcoholic group. In the Dionysos study, obesity among heavy drinkers, increased the risk for steatosis by twofold [19]. Moreover, no significant differences were evident concerning conditions associated with metabolic syndrome neither at baseline nor at follow-up, but we had expected a higher portion of diseases associated with metabolic syndrome in the NAFLD group. In a study from Denmark, a significantly higher BMI was seen in NAFLD than in AFLD patients. However, this might at least partly reflect the fact that their patients were recruited from an obesity research project [8] whereas our patients were unselected patients undergoing a liver biopsy.

In the current study women were in the majority in the NAFLD group but the high proportion of women with fatty liver compared to men may reflect a higher disease burden in women. A recent study from the US also found a higher proportion of women in the NAFLD group [22] which is in line with our results. A significantly higher prevalence of cirrhosis in female AFLD patients was observed in a Danish study and time to cirrhosis was associated with female gender [10]. Population based studies have shown increased risk of women developing alcohol-induced cirrhosis [23–26].

Progression of NAFLD has been found to be slow and seems to depend a great deal on the initial fibrosis stage [8, 14]. Patients with simple fatty liver at baseline seem to have a good prognosis in terms of liver disease. In a Danish study of 109 patients diagnosed with pure non-alcoholic simple steatosis (without inflammation or significant fibrosis) only one of the patients developed cirrhosis [8]. In the current study more severe lobular inflammation was found in the AFLD group compared to the NAFLD group and a significantly higher number of patients in the AFLD group had steatohepatitis compared to the NAFLD group.

In the total study cohort patients with more severe fibrosis at baseline showed a worse overall survival than patients with none or mild fibrosis at baseline. Based on this we were able to show an association in the total study cohort between the stage of fibrosis and the prognosis. However more NAFLD than AFLD patients had mild or no fibrosis at baseline. This is in agreement with results from a recent study showing that advanced fibrosis in the index liver biopsy was the most important predictor of the prognosis in these patients [27]. A recent Danish study showed that the cirrhosis risk was more than twice as high for the patients with steatohepatitis than for those with pure steatosis [26].

A Swedish cohort study of patients with biopsy-proven NAFLD and elevated liver tests showed that they had a similar survival compared to the Swedish population [7]. Interestingly the risk of death was increased in patients with non-alcoholic steatohepatitis [7].

A study from Minnesota, identified 420 patients with NAFLD by imaging or liver biopsy found liver-related complications to be the third most common cause of death among NAFLD patients [6]. This is similar to our results showing liver-related to be the third most common cause of death amongst the NAFLD group. However in the AFLD group liver-related death was the leading cause of death, followed by cardiovascular diseases and malignancy which is in accordance to a previous study where hepatobiliary disease was the leading cause of death in the AFLD [9].

In the current study, no patient in the NAFLD group died of hepatocellular carcinoma and only one patient in the AFLD group. The absence of HCC among the NAFLD group differs from previous cohort studies showing that 3/129 (2%) [7] and 2/420 (0.5%) [6] of NAFLD patients developed HCC [7]. A reasonable explanation for this difference in our study could be that our patients had in general mild changes in the liver biopsy at baseline. It is also conceivable that a longer follow-up time would probably lead to patients with HCC.

### Cardiovascular disease

In agreement with many previous studies a markedly higher proportion of our women in the NAFLD group died of cardiovascular disease compared to women in the AFLD group, 17 patients vs. one patient respectively. A previous study [10] showed that cardiovascular disease was the leading cause of death in the AFLD group (men and women toghether), which is at odd with our results. In the current study the leading cause of death in the AFLD group was liver related. We can not find a plausible explanation for this difference, although it has been shown that diagnoses on death certificates can underestimate liver-related mortality [28] which might have been the case in the Danish study [10].

The most common cause of death in the NAFLD group was from cardiovascular disease, followed by malignancy which is in agreement with findings of other cohort studies (6, 7, 14, 16,). One study found 34% increased risk of cardiovascular mortality among NAFLD patients compared to the general Minnesota population over a 7.6 year follow-up [6].

## Conclusion

In conclusion a higher proportion of patients with AFLD developed liver cirrhosis and had liver-related death compared to patients with NAFLD in this population based setting and had also more severe histological changes in the liver biopsy at baseline**.** Patients in the AFLD group showed a significantly worse survival compared to patients in the NAFLD group. Patients with more severe fibrosis at baseline showed a worse survival than patients with none or mild fibrosis at baseline. Patients with fatty liver disease showed a markedly higher risk of developing liver-related death compared to the general population.

## Abbreviations

NAFLD: Nonalcoholic fatty liver disease
AFLD: Alcoholic fatty liver disease
HR: Hazard ratio
SNOMED: Systemized nomenclature of medicine
NUH: National university hospital
RCD: Registry of causes of death
PAS: Periodic acid schiff
ALT: Alanine aminotransaminase
AST: Aspartate aminotransferase
ALP: Alkaline phosphatase
PT: Prothrombin time
PLT: Platelets count
TG: Triglycerides
MCV: Mean corpuscular volume
NS: Non significant
NASH: Non-alcoholic steatohepatitis
ASH: Alcoholic steatohepatitis
NAS: Non-alcoholic fatty liver disease activity score
HCC: Hepatocellular carcinoma
BMI: Body mass index.

## References

[1] Bellantini S, Scaglioni F, Marino M, Bedogni G (2010). Epidemiology of non-alcoholic fatty liver disease. Dig Dis.

[2] Lazo M, Clark JM (2008). The epidemiology of nonalcoholic fatty liver disease: a global perspective. Semin Liver Dis.

[3] Angulo P (2002). Nonalcoholic fatty liver disease. N Engl J Med.

[4] Friis-Liby I, Aldenborg F, Jerlstad P, Rundström K, Björnsson E (2004). High prevalence of metabolic complications in patients with non-alcoholic fatty liver disease. Scand J Gastroenterol.

[5] Day PC (2002). Non-alcoholic steatohepatitis (NASH): where are we now and where are we going. Gut.

[6] Adams LA, Lymp JF, St. Sauver J, Sanderson SO, Lindor KD, Feldstein A, Angulo P (2005). The natural history of nonalcoholic fatty liver disease: a population-based cohort study. Gastroenterology.

[7] Ekstedt M, Franzén LE, Mathiesen UL, Thorelius L, Holmqvist M, Bodemar G, Kechagias S (2006). Long-term follow-up of patients with NAFLD and elevated liver enzymes. Hepatology.

[8] Dam-Larsen S, Franzmann M, Andersen IB, Christoffersen P, Jensen LB, Sörensen TIA, Becker U, Bendtsen F (2004). Long term prognosis of fatty liver: risk of chronic liver disease and death. Gut.

[9] Jepsen P, Vilstrup H, Mellemkjær L, Thulstrup AM, Olsen JH, Baron JA, Sörensen HT (2003). Prognosis of patients with a diagnosis of fatty liver – a registry-based cohort study. Hepato-Gastroenterology.

[10] Dam-Larsen S, Becker U, Franzmann MB, Larsen K, Christoffersen P, Bendtsen F (2009). Final results of long-term, clinical follow-up in fatty liver patients. Scand J Gastroenterol.

[11] Kleiner DE, Brunt EM, Van Natta M, Behling C, Contos MJ, Cummings OW, Ferrell LD, Liu YC, Torbenson MS, Unalp-Arida A, Yeh M, McCullough AJ, Sanyal AJ (2005). Design and validation of a histological scoring system for nonalcoholic fatty liver disease. Hepatology.

[12] Brunt EM, Janney CG, Di Bisceglie AM, Neuschwander-Tetri BA, Bacon BR (1999). Nonalcoholic steatohepatitis: a proposal for grading and staging the histological lesions. Am J Gastroenterol.

[13] Statistics Iceland 2013.http://www.hagstofan.is/Hagtolur/Mannfjoldi/faeddir-og-danir

[14] Matteoni CA, Younossi ZM, Gramlich T, Boparai N, Liu YC, McCullough AJ (1999). Nonalcoholic fatty liver disease. A spectrum of clinical and pathological severity. Gastroenterology.

[15] Ong JP, Pitts A, Younossi ZM (2008). Increased overall mortality and liver-related mortality in non-alcoholic fatty liver disease. J Hepatol.

[16] Söderberg C, Stål P, Askling J, Glaumann H, Lindberg G, Marmur J, Hultcrantz R (2010). Decreased survival of subjects with elevated liver function tests during a 28-year old follow up. Hepatology.

[17] Lee GR (1989). Nonalcoholic steatohepatitis: a study of 49 patients. Hum Pathol.

[18] Propst A, Propst T, Zangerl G, Ofner D, Judmaier G, Vogel W (1995). Prognosis and life expectancy in chronic liver disease. Dig Dis Sci.

[19] Bellantini S, Saccoccio G, Masutti F, Croce LS, Brandi G, Sasso F, Cristanini G, Tiribelli C (2000). Prevalence of and risk factors for hepatic steatosis in Northern Italy. Ann Intern Med.

[20] Naveau S, Giraud V, Borotto E, Aubert A, Capron F, Chaput J (1997). Excess weight risk factor for alcoholic liver disease. Hepatology.

[21] Gunnarsdottir SA, Olsson R, Olafsson S, Cariglia N, Westin J, Thjodleifsson B, Björnsson E (2009). Liver cirrhosis in Iceland and Sweden: incidence, aetiology and outcomes. Scand J Gastroenterol.

[22] Neuschwander-Tetri BA, Clark JM, Bass NM, Van Natta M, Unalp-Arida A, Tonascia J, Zein CO, Brunt EM, Kleiner DE, McCullough AJ, Sanyal AJ, Diehl AM, Lavine JE, Chalasani N, Kowdley KV (2010). Clinical, laboratory and histological associations in adults with nonalcoholic fatty liver disease. Hepatology.

[23] Klatsky AL, Armstrong MA, Friedman GD (1992). Alcohol and mortality. Ann Intern Med.

[24] Becker U, Deis A, Sorensen TIA, Borch-Johnsen K, Muller CF, Schnohr P, Jensen G (1996). Prediction of risk of liver disease by alcohol intake, sex, and age: a prospective population study. Hepatology.

[25] Kamper-Jorgensen M, Gronbaek M, Tolstrup J, Becker U (2004). Alcohol and cirrhosis: dose–response or threshold effect?. J Hepatology.

[26] Deleuran T, Grønbæk H, Vilstrup H, Jepsen P (2012). Cirrhosis and mortality risks of biopsy-verified alcoholic pure steatosis and steatohepatitis: a nationwide registry-based study. Aliment Pharmacol Ther.

[27] Angulo P, Bugianesi E, Björnsson E, Charatcharoenwitthaya P, Mills PR, Barrera F, Haflidadottir S, Day CS (2013). Simple non-invasive systems predict long-term outcomes in patients with nonalcoholic fatty liver disease. Gastroenterology.

[28] Prytz H, Skinhoj P (1981). Morbidity, mortality, and incidence of cirrhosis in Denmark 1976–1978. Scan J Gastroent.

[CR29] The pre-publication history for this paper can be accessed here:http://www.biomedcentral.com/1471-230X/14/166/prepub

